# Semi-automated 3DED/microED data collection and processing unveil multiple polymorphs in biogenic guanine crystals from fish

**DOI:** 10.1063/4.0001076

**Published:** 2025-10-27

**Authors:** Naruhiko Adachi

**Affiliations:** University of Tsukuba, Tsukuba, Ibaraki, Japan

## Abstract

Certain fish exhibit silver or blue color not due to pigmentation, but as a result of specialized guanine crystals embedded within their skin. These crystals are located in cells termed iridophores and are organized in stratified layers that reflect incident light. Irregular interlayer spacing results in a silvery appearance, whereas uniform spacing produces a blue hue. The phenomenon of structural coloration in fish has attracted considerable scientific interest due to its potential applications in advanced materials, including cosmetics and functional surface coatings. However, the extremely small size of these crystals—typically at the micrometer scale—poses considerable challenges for detailed structural analysis.

Guanine crystals are known to exist in multiple polymorphic forms. Single-crystal X-ray diffraction (SC-XRD) analyses of synthetic guanine, alongside powder X-ray diffraction (PXRD) analysis of biogenic guanine, suggest that fish-derived crystals adopt the anhydrous α- and/or β- polymorphs. Recent advances in 3DED/microED techniques have demonstrated that guanine crystals obtained from salmon correspond to the β- polymorph. Nevertheless, the specific crystalline architectures employed by other fish species remain largely unresolved—particularly in species capable of dynamic color modulation. The exceptionally small dimensions of these crystals further complicate their isolation and render them highly susceptible to electron beam-induced damage. Consequently, the diffraction data from hundreds to thousands of individual microcrystals is required to achieve reliable structural determination, and automating the microED data collection and processing workflow is imperative.

In this study, we developed a semi-automated microED data collection and processing system employing the CRYOARM200-Rio-SerialEM configuration. Initial validation was conducted using synthetic guanine crystallized under various conditions, from which diffraction data were collected from 143 crystals formed under acidic conditions (corresponding to the monohydrate form) and 216 crystals formed under basic conditions (corresponding to the anhydrous α- and β-polymorphs). Subsequent structural analyses of 506 guanine crystals from Pacific saury, 651 from Pacific cutlassfish, and 2,127 from blue damselfish revealed that in addition to the anhydrous β-polymorph, guanine crystals from these species also exhibit the anhydrous α-polymorph. These findings are consistent with previous PXRD observations. Collectively, our results validate the effectiveness of our semi-automated microED pipeline in resolving structural heterogeneity within micrometer-scale biogenic crystals.

